# Long-term longitudinal study on swine VML model

**DOI:** 10.1186/s13062-023-00399-1

**Published:** 2023-07-31

**Authors:** Francesca De Paolis, Stefano Testa, Gabriele Guarnaccia, Alessio Reggio, Ersilia Fornetti, Felice Cicciarelli, Rebecca Deodati, Sergio Bernardini, Daniele Peluso, Jacopo Baldi, Roberto Biagini, Flavia Cobianchi Bellisari, Antonio Izzo, Ferruccio Sgalambro, Francesco Arrigoni, Francesco Rizzo, Stefano Cannata, Tommaso Sciarra, Claudia Fuoco, Cesare Gargioli

**Affiliations:** 1 Department of Biology, University of Rome “Tor Vergata”, Rome, 00133 Italy; 2 PhD Program in Cellular and Molecular Biology, Department of Biology, University of Rome “Tor Vergata”, Rome, Italy; 3 Marseille Medical Genetics, Aix-Marseille University, INSERM, Marseille, MMG France; 4 IRCCS Regina Elena National Cancer Institute, Rome, Italy; 5 Department of Clinical Sciences and Applied Biotechnologies (DISCAB), Aquila, Italy; 6 Joint Veteran Center, Scientific Department, Army Medical Center, Rome, Italy

**Keywords:** Skeletal muscle, Volumetric muscle loss, Scar tissue, Vascularization, Inflammation

## Abstract

**Background:**

Volumetric Muscle Loss (VML), resulting from severe trauma or surgical ablation, is a pathological condition preventing myofibers regeneration, since skeletal muscle owns the remarkable ability to restore tissue damage, but only when limited in size. The current surgical therapies employed in the treatment of this pathology, which particularly affects military personnel, do not yet provide satisfactory results. For this reason, more innovative approaches must be sought, specifically skeletal muscle tissue engineering seems to highlight promising results obtained from preclinical studies in VML mouse model. Despite the great results obtained in rodents, translation into human needs a comparable animal model in terms of size, in order to validate the efficacy of the tissue engineering approach reconstructing larger muscle mass (human-like). In this work we aim to demonstrate the validity of a porcine model, that has underwent a surgical ablation of a large muscle area, as a VML damage model.

**Results:**

For this purpose, morphological, ultrasound, histological and fluorescence analyses were carried out on the scar tissue formed following the surgical ablation of the *peroneus tertius* muscle of *Sus scrofa domesticus* commonly called mini-pig. In particular, the replenishment of the damaged area, the macrophage infiltration and the vascularization at different time-points were evaluated up to the harvesting of the scar upon six months.

**Conclusion:**

Here we demonstrated that following VML damage, there is an extremely poor regenerative process in the swine muscle tissue, while the formation of fibrotic, scar tissue occurs. The analyses performed up to 180 days after the injury revealed the development of a stable, structured and cellularized tissue, provided with vessels and extracellular matrix acquiring the status of granulation tissue like in human.

**Supplementary Information:**

The online version contains supplementary material available at 10.1186/s13062-023-00399-1.

## Introduction

Skeletal muscle is one of the most copious tissue in the human body and it is able to self-regenerate after tissue damage. This regenerative process is mediated by resident progenitor cells:

- satellite cells (SCs), by playing a dominant role in maintaining homeostasis and muscle regeneration [1], are able to activate themselves in response to physiological and pathological stimuli, giving rise to myoblasts capable of fusion and differentiation for myofiber recovery [2];
- fibro-adipogenic progenitors (FAPs), important for myogenic cells differentiation induction [3, 4];
- stem cells associated with blood vessels, such as mesoangioblasts (MABs) or pericytes, contractile and multipotent mesenchymal cells, that tightly envelop the blood vessels endothelium [5–7];
- muscle progenitor cells resident in the interstitial spaces (PICs), present among muscle fibers and characterized by the expression of the cellular stress mediator Pw1 [8];
- more recently isolated cells belonging to the Side Population (SP), associated with the endothelium and the outermost lining of the vessels[9, 10].
- no less important are the fibroblasts, that upon a muscle injury, are recruited from the surrounding connective tissue to the damage site, differentiating into myofibroblast (MFs) [11]. It has been demonstrated during an aberrant regeneration, that MFs counteract the SCs functionality by inhibiting their proliferation [12], then leading to an impairment in the muscle repair and an increasing in the muscle fibrosis.

Indeed, the regenerative capacity of the skeletal muscle tissue is limited by the extent of the damage: massive damage leads to permanent loss of muscle mass that reduces, or in the worst case, totally compromises muscle function[13–15]. This disabling pathological condition, known as Volumetric Muscle Loss (VML), can occur following serious primary trauma (such as crash injuries, blasts or penetrating injuries) or as a consequence to sarcomas surgical ablation [16]. Following VML, the natural regenerative abilities of skeletal muscle tissue are lost due to a massive depletion of stem cells and extracellular matrix [17]. Among the different currently available treatments for VML, non-invasive therapies are based on the maximization of the remaining muscle strength, while surgical interventions usually prefer a conservative approach rather than amputation and consist in the transplantation of free muscle flaps transferred from a donor to a recipient area [18]. Unfortunately, the obtained results are unsatisfactory both in renovating muscle mass and function [15].

To address the limits of the currently employed therapies, a keen interest in developing more innovative and effective approaches has grown in the scientific community. In particular, skeletal muscle tissue engineering produced promising results in preclinical studies on VML mouse model, revealing the capability to restore muscle mass [19, 20]. A further improvement has been reached with the introduction of the 3D-bioprinting technology, which allows to obtain cellularized and highly organized biological substitutes in which the native skeletal muscle tissue architecture is reproduced [21, 22]. However, despite the promising results, this approach cannot be directly translated to human therapy due to the significant increase in muscle tissue size. Therefore, in order to demonstrate the efficacious scaling-up of tissue engineering technology, the characterization of an experimental model comparable to human size, is fundamental. Particularly, because of the similarity to humans in size, physiology, and genetics, pig has already demonstrated to be an excellent choice as an animal model for evaluating tissue engineering effectiveness [23]. As for the VML study, in previous works employing porcine models, functional testing, histological and gene expression analyses were conducted at a maximum experimental time point of 120 days, confirming the inability of skeletal muscle to regenerate after a large damage [24–26].

Nevertheless, to date, a detailed long-term study of the repair process progression upon VML in a large-scale animal model is still missing.

In this work, we present a longitudinal study to validate a swine model subjected to a large muscle area surgical ablation, as a model of VML. For this purpose, morphological, ultrasound and histological analyses were carried out on the scar tissue formed following the surgical ablation of *Sus scrofa domesticus peroneus tertius* muscle. Obtained data highlighted the complete lack of muscle fibers in the repair tissue, a progressive decrease of the macrophage infiltrate at later time points and the final formation of a stable and vascularized granulation/scar tissue.

## Results

### Experimental set-up of the VML model production

The term VML indicates a partial ablation of muscular tissue that since cannot be endogenously regenerated leads to a persistent functional deficit of the muscle [27].

Preclinical VML models have been created in several species, including mice [28–30], rats [30–34], dogs[35] and pigs[26, 36] in which a sterile surgical mechanism of injury has been used to develop the trauma.

In all these models the removal of at least 20% of the tissue has been considered as the minimal value necessary to generate the injury. Indeed, such damage seems to overwhelm the capability of regeneration, leading to the deposition of non-contractile scar tissue at the defect site. As a result, there is a chronic loss of muscle and limb function [15, 37].

**Fig. 1:**
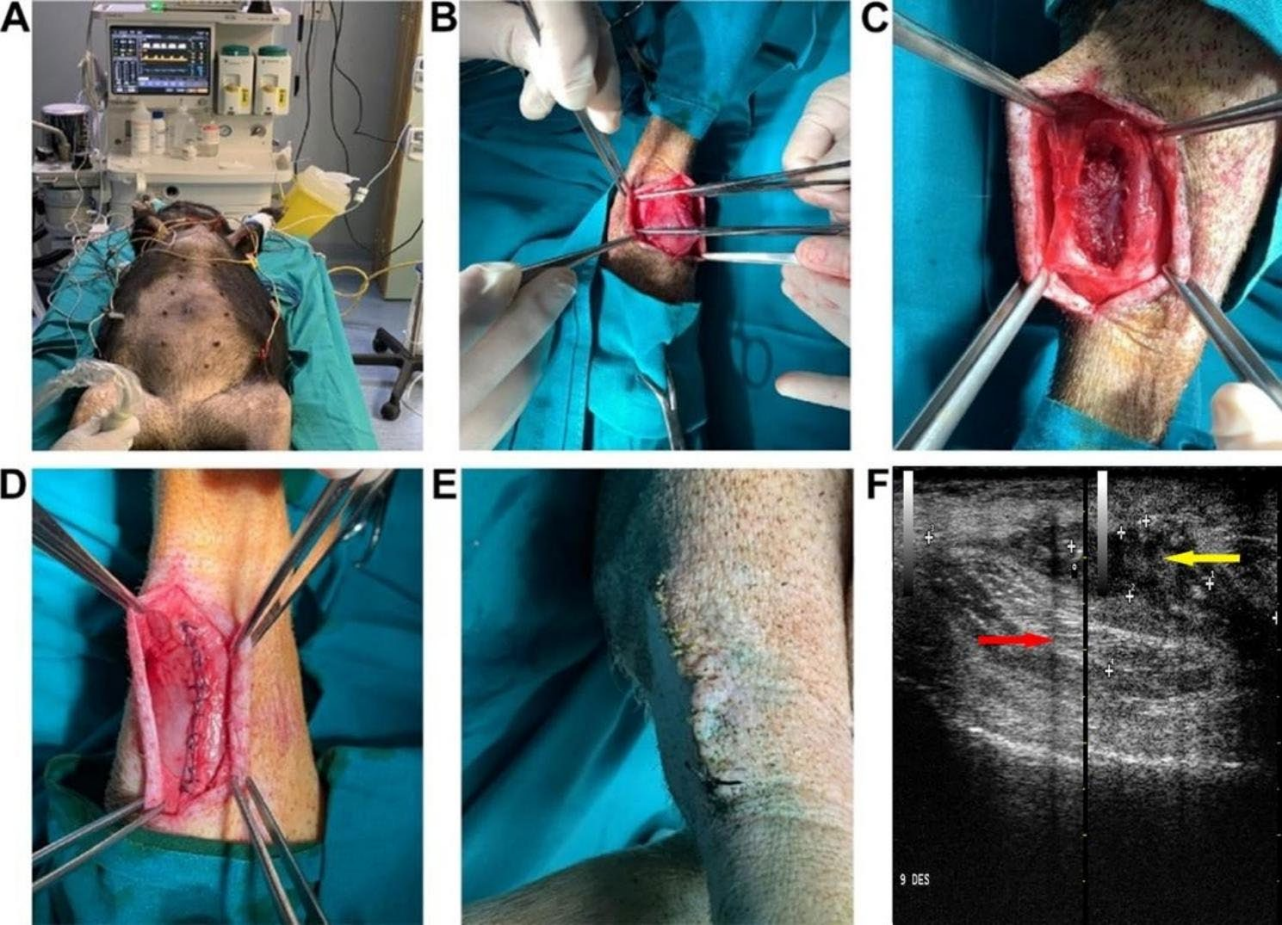
**Surgical procedure performed to produce a porcine model of VLM.** (**A**). The operation was carried out in a surgical room under the supervision of veterinarian anesthesiologists under constant instrumental monitoring of the animals. (**B-D**). Muscle ablation and saturation of the lesion. (**E**). Representative images referring to scar monitoring after 15 days from the damage. (**F**). Representative ultra-sound image showing tissue organization of the muscle lesion observed 15 days from the damage. The yellow and the red arrows indicate respectively the muscle ablated zone and the muscle fibers

Therefore, to generate a VML swine model in accordance with the previous works performed by Ward et al., 2016 and Greising et al., 2017 we surgically removed the 27,2% ± 6,2%, of *Peroneus tertius* muscle in *Sus scrofa domesticus* animals. Afterwards, the lesion was sutured and monitored starting from 15 days up to 180 days. At 15 days, the wound did not show any alterations, ultrasound analyses were performed accordingly to observe the tissue organization at the muscle lesion. The daily monitored scar has not undergone any alterations and the ultra-sound images, acquired 15 days after the lesion, revealed the damaged area as a black area between perfectly intact and ordered muscle bundles with white appearance (Fig. 1).

### Ultrasound muscle lesion: monitoring and quantification of injury replacement over time

**Fig. 2:**
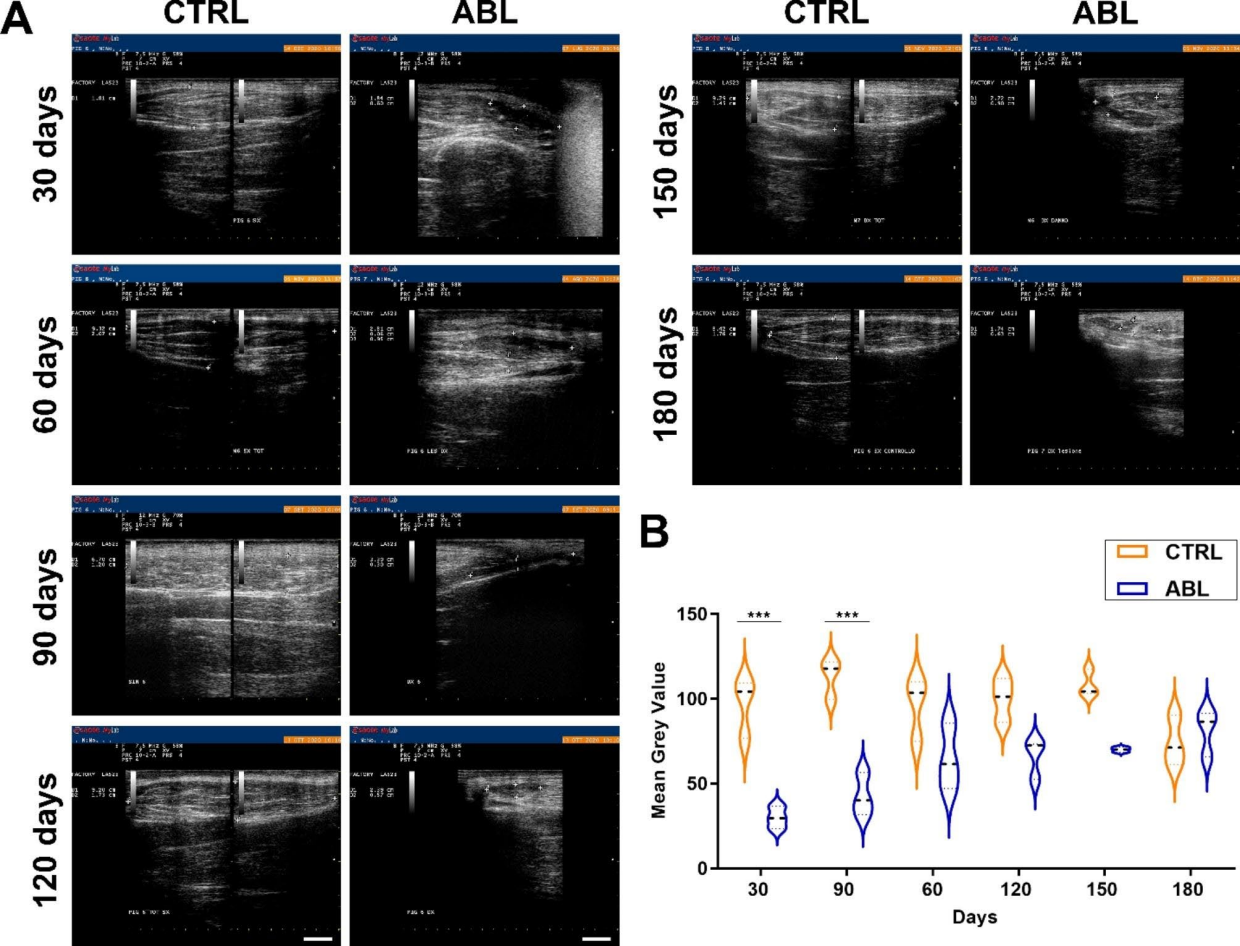
**Ultrasound examination after* Peroneus tertius* muscle surgical removal (3 × 2 × 1 cm) (n = 5)**. (**A**). Representative eco-graphic images of ablated (ABL) and healthy (CTRL) muscles analyzed at 30, 60, 90, 150 and 180 days after ablation. The removed zone appears as a black area delimited in the pictures by white crosses, while the orderly muscle fibers populate the white area. (**B**). Violin plot showing the Mean Grey Value calculated from ultrasound images for each time points to evaluate lesion filling. The violin plot quantitated the sum of the gray values of all the pixels in the selection divided by the number of pixels: reported in calibrated units (e.g., optical density). For each time point n = 10 ultrasound images were analyzed (n = 2 for each animal) and the average value of the MGV was graphed. One way ANOVA Tukey’s Multiple Comparison test was performed. Scale bars: CTRL = 2 cm; ABL = 1 cm.

The extent of muscle damage, the dimension stability and the tissue features were characterized by ultrasound examinations that were carried out at 30, 60, 90,150 and 180 days after muscle ablation. In particular, aligned muscle bundles resulted opaque to ultrasounds (hyperechoic, white) on the contrary of the lesions that appeared, between the fibers, as empty spaces transparent in ultrasounds (anechoic, black) (Fig. 2). We noticed that the lesions were filled during the time, probably thanks to the activation of resident cells that already at 60 days after the muscle lesion, were able to replace the damage, producing a tissue with a disorganized and granular appearance.

In order to obtain a quantitative evaluation of the injury area replacement, the ultrasound images from different experimental time points, were used to assess the Mean Grey Value (MGV) that was calculated in control, undamaged, muscle and at the ablation site. MGV, greater in correspondence of tissues that are lighter for the ultrasound opacity, resulted significantly increased in time dependent manner (Fig. 2B), confirming the formation of scar tissue.

### Morphological and histological profiling of the repairing tissue following the VML damage

**Fig. 3:**
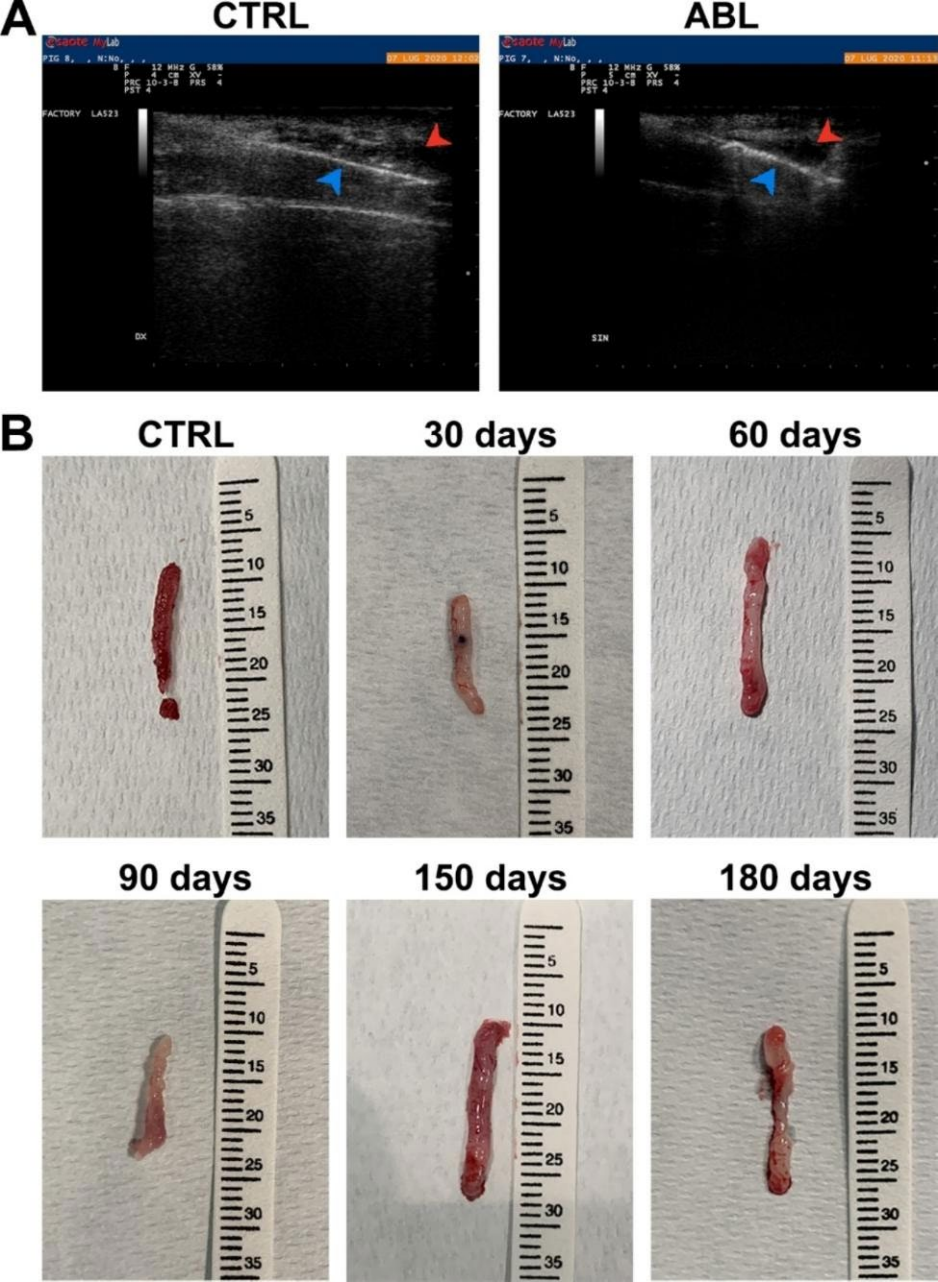
**Muscle biopsies taken from 30 up to 180 days after ablation**. (**A**). Representative images of eco-guided ultrasounds showing the needles at the site of the muscle, where the biopsies had been performed in the CTR and ABL animals. The presence of the needle is indicated by the blue and red arrow heads. (**B**). Representative images of biopsies derived from CTR and damaged muscle (30, 60, 90, 150 and 180 days after injury). We reported the biopsy derived from undamaged muscle (CTRL) just in a representative image

Muscle biopsies were obtained to analyze from a morphological and histological point of view the repairing tissue over time. In order to minimize the variability due to a removal of undamaged tissue, the needle biopsies were picked-up by ultrasound-guiding technique (Fig. 3A). To have a first macroscopic examination of the isolated tissue, biopsies were photographed at each time point of the conducted analysis revealing a striking difference in consistency and coloring of the samples, taken from ablated *Peroneus tertius* compared with the control one. The presence in the biopsies derived from the injured area, of white and compact tissue portions, was an index of fibrotic and fatty tissue deposition replacing the removed myofibers (Fig. 3B).

**Fig. 4:**
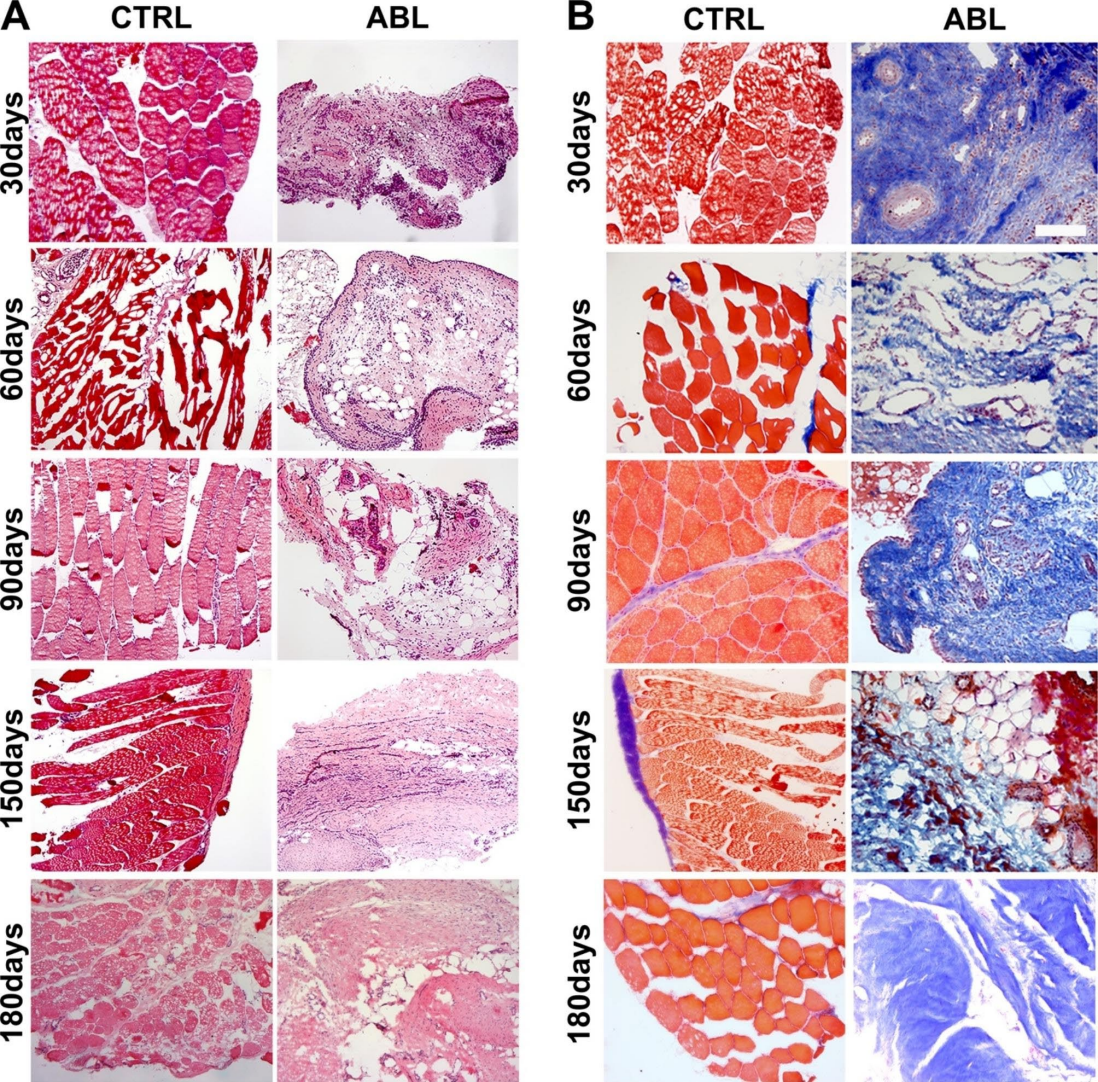
**Morphological and histological characterization of healthy and damaged muscle tissue.** Representative images of hematoxylin and eosin (H&E) (**A**) and Masson’s trichrome staining (**B**) of biopsies derived from uninjured and ablated muscles at 30, 60, 90, 150 and 180 days after muscle damage. All along the considered time points the fibrotic tissue is marked in blue. 20X magnification; scale bar: 100 μm

Thereafter, tissue sections were labeled with hematoxylin eosin (H&E) (Fig. 4A) and Masson’s trichrome (Fig. 4B) staining to have a quick and deep evaluation of the observed tissue over time. Sections of the uninjured tissue biopsies were characterized by muscle tissue where the muscle fibers were well identifiable and organized in structured tissue. On the contrary, the sections obtained from the repairing tissue generated upon muscle ablation, showed an unstructured appearance, enriched in collagen and totally devoid of skeletal muscle fibers, compatible with a granular, scar, tissue of fibrotic nature.

### Evaluation of inflammatory infiltrates in the scar tissue

**Fig. 5:**
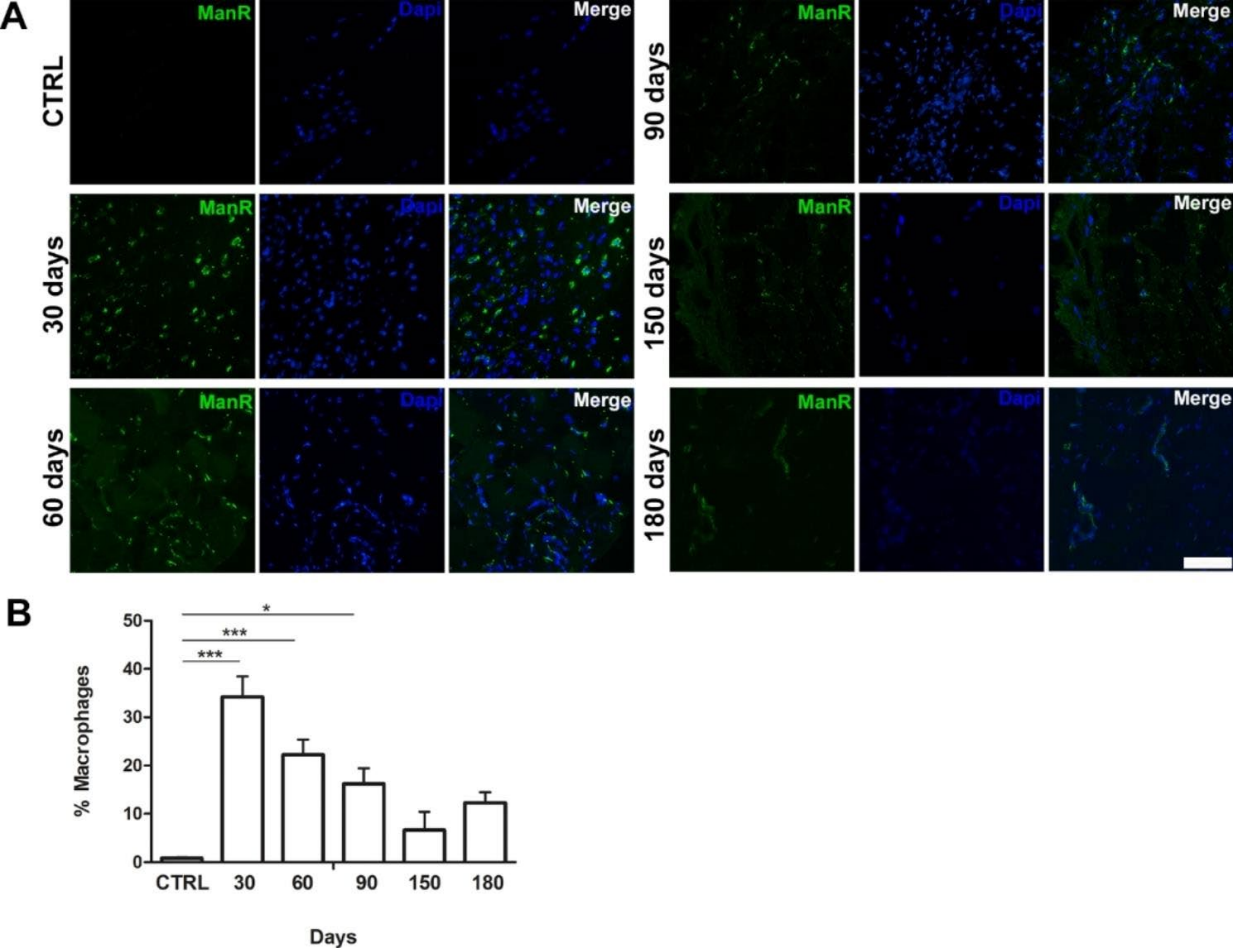
**Evaluation of inflammatory infiltrates.** (**A**) Immunofluorescence analysis of the inflammatory infiltrates in the ablated tissue at different time points (30, 60, 90, 150 and 180 days) compared to the control. Scale bar 50 μm. (**B**) Bar plot showing the percentage of positive area for ManR obtained through the ImageJ program (n = 20 ± 2 images for each experimental point). One way ANOVA Tukey’s Multiple Comparison test was performed

To analyze the level of inflammation at the ablation site, immunofluorescences analyses were performed to reveal the mannose receptor (also known F4/80): a glycoprotein commonly expressed by macrophages (Fig. 5A). From the quantitative evaluations carried out on the obtained images, it is evident how the macrophage infiltration was, up to 90 days after the muscle lesion, significantly higher than the control, contemporarily showing a trend of decreasing (about 20%) starting from the time point of 60 days, reaching low levels 150 days after muscle ablation (Fig. 5B). Even if the rate of macrophage scored was higher than that of the previous time point at 180 days after lesion, it remained lower compared to the other time points and not statistically significant compared to the control.

### Vascularization analysis

**Fig. 6:**
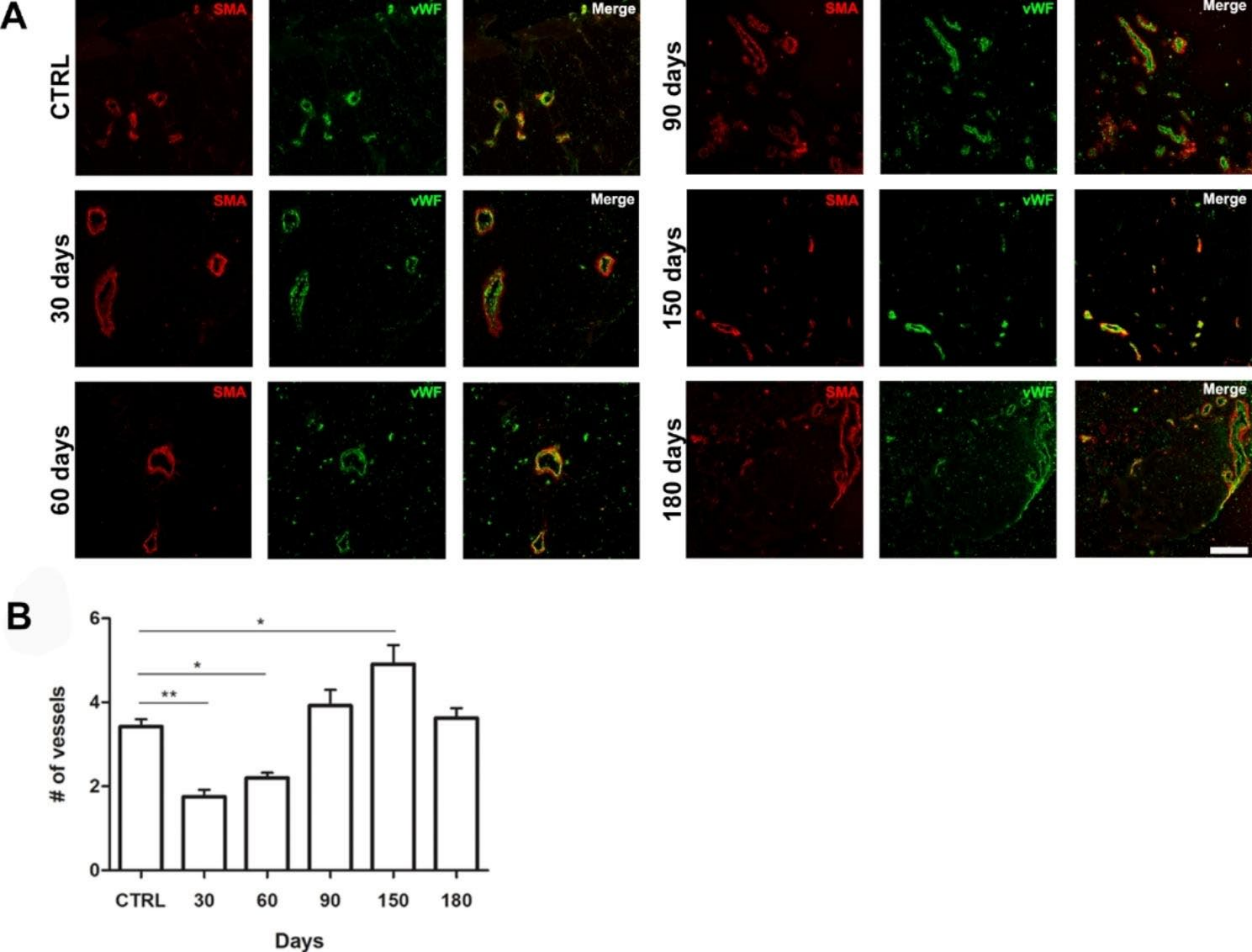
**Vasculogenesis process evaluation.** (**A**) Immunofluorescence analysis of the vascularization in the ablated tissue at different time points (30, 60, 90, 150 and 180 days) compared to the control. Scale bar: 100 μm. (**B**) Bar plot showing the number of vessels positive for α-SMA (n = 20 ± 2 images for each experimental point). One way ANOVA Tukey’s Multiple Comparison test was performed

Vasculogenesis process was investigated by immunofluorescence analysis to evaluate the formation of new blood vessels during the reconstructive events. In particular, they were identified on scar tissue sections evaluating the expression of Alpha Smooth Muscle Actin (α-SMA) and Von Willebrand Factor (vWF), two of the main markers used to respectively reveal the muscle structure and the vessels endothelium (Fig. 6A). The obtained fluorescence signals were analyzed through the imageJ software to count the α-SMA positive vessels. In particular, they were quantified by comparing the control (CTRL) with the ablated samples at different time points (30, 60, 90, 150 and 180 days). The ablated samples showed an increasing trend in vessels number over time reaching its peak at 150 days after ablation. In particular, even if an increased trend was seen respectively at the time points of 30 and 60 days, the number of vessels was significantly lower than the control, becoming the increment statistically significant only at the time point of 150 days. At the end of the reparative process, 180 days after ablation (six months after the muscle damage), the vessels number returned to be similar to those of the control suggesting that although muscle recovery was not observed, the missing tissue was replaced by a fibrous and vascularized tissue (Fig. 6B).

### Characterization of the scar tissue deposited after the VML damage

**Fig. 7:**
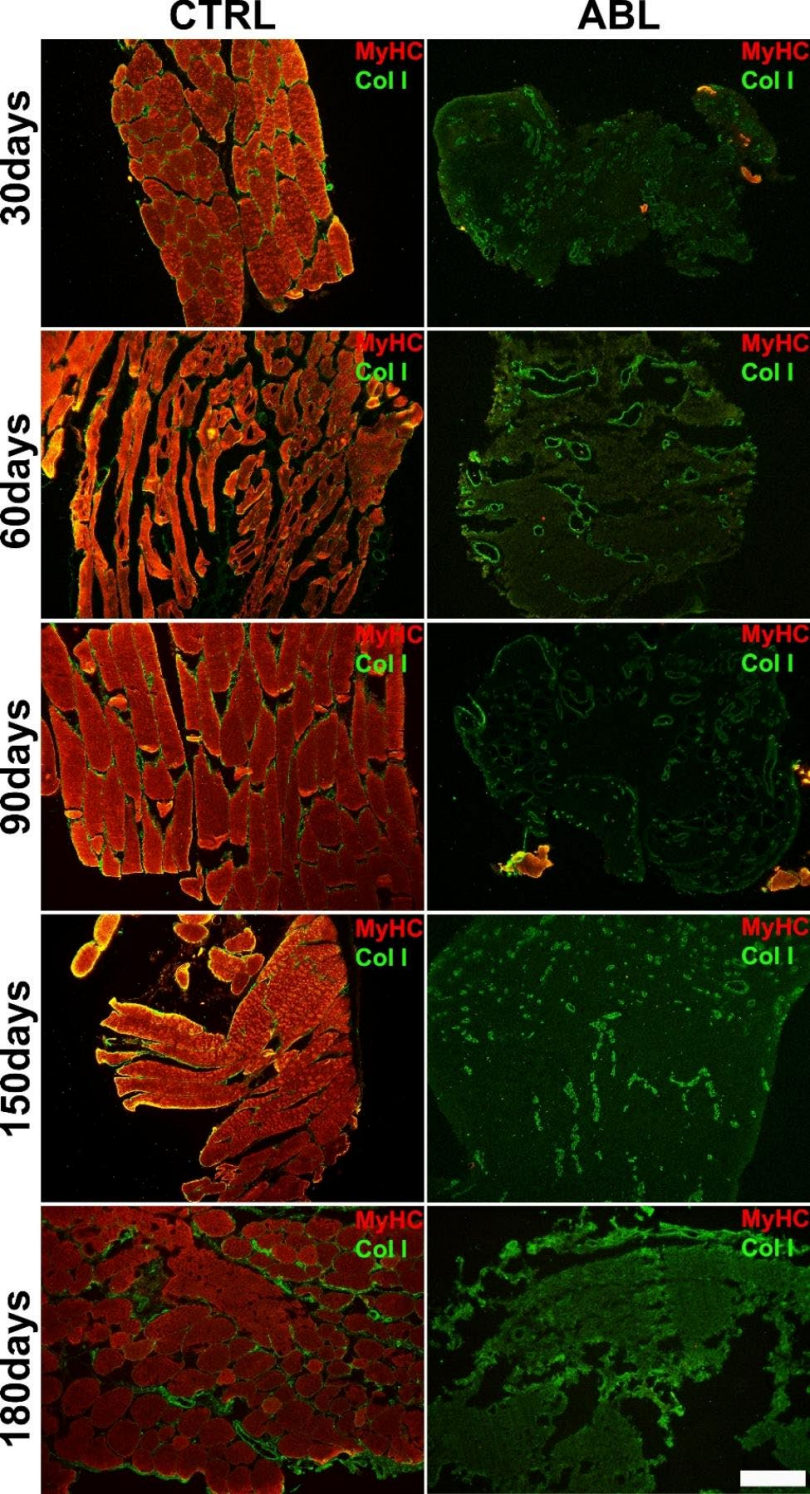
**Deep characterization of scar tissue**. Immunofluorescence analysis for the expression of MyHC and ColI in the ablated tissue (ABL) at different time points (30, 60, 90, 150 and 180 days) compared with the control (CTRL). Scale bar: 100 μm

To analyze whether the muscle reparative process after VML damage, was able to generate new muscle fibers autonomously, the needle biopsy sections were stained to reveal by means of immunofluorescence analysis the expression of the Sarcomeric Myosin Heavy Chain (MyHC). In addition, to have a complete picture, the samples were further labeled with type I Collagen (ColI) to reveal fibrous infiltration (Fig. 7). The obtained results, highlight that the muscle was unable to regenerate muscle fibers since the *Peroneus tertius* after damage showed exclusively granular and scar tissue, positive for ColI.

## Discussion

Skeletal muscle is a tissue playing a pivotal role in numerous and different activities of daily life, such as breathing, walking, speaking and writing.

Due to its continuous activity and its superficial localization, skeletal muscle tissue is prone to injury [38]. Traumatic events of various nature, such as excessive physical exertion, surgical ablations, accidents or degenerative pathologies, cause muscle damage of different severity, which, if not adequately repaired, can produce loss of muscle mass, sometimes to an extent that leads to partial or total impairment of tissue function, namely VML (Volumetric Muscle Loss). By virtue of the dramatic impact that this event has on the physical and psychological conditions of patients suffering from this condition, VML is now being studied for the development of a skeletal muscle reconstructive therapy [14, 39]. Skeletal muscle tissue is, in fact, able to regenerate itself autonomously for normal tissue turnover and following small lesions, thus preserving its integrity. It is, therefore, necessary to develop effective clinical therapies representing valid tools for the recovery of muscle tissue in the event of large damages. Despite regenerative medicine has already provided very satisfactory results in the reconstruction of different tissues such as skin, bone, cartilage, trachea and bladder [40–44], for skeletal muscle tissue is still an open challenge.

New skeletal muscle tissue engineering approaches are continuously applied with more or less success in studies conducted on VML mouse models [7, 19]. However, the transition from rodents to humans, is linked to a significant increase in the size of muscular tissue that the engineered constructs must be able to recover. It is, therefore, necessary to validate the effectiveness of tissue engineering in an animal model with muscle dimensions comparable to human ones.

In this regard, we generated a swine VML model for large muscle size reconstruction by 3D bio-printing approach, opening the opportunity to translate this approach into human therapy, given the comparable muscle dimensions.

Since little is known about the repair process progression upon VML in a large-scale animal, in this study we presented a detailed long-term evaluation of all main events involved in the formation of the repairing tissue.

Thus, we focused on the characterization of reparative tissue formed after surgical ablation (3 × 2 × 1 cm) of the *Peroneus tertius* muscle in a porcine model of VML.

Initially, ultrasound testing were carried out revealing a substantial difference between healthy and ablated muscle. In fact, after 90 and 150 days from the injury, the ablation site displayed a different density compared to the surrounding muscular tissue, suggesting fibrotic infiltration filling the empty area and lacking of muscle regeneration at the ablation site. The eco-guided needle biopsies performed confirmed the non-muscular nature of the tissue given the white color and very compact appearance, subsequently confirmed by the histological analysis. Indeed, the hematoxylin-eosin and in particular Masson’s trichrome staining confirmed the different nature of the reparative tissue, highlighting its fibrotic nature.

The level of macrophage infiltrate was studied to evaluate the inflammatory response, showing a continuous decrease until returning to be comparable with the level measured in the undamaged control muscle. Only at 180 days we have a slight increase in the macrophagic infiltrate but in any case, the difference is not statistically significant compared to the control, demonstrating in the inflammation decreasing.

Subsequently the vascularization of the ablated area at different time points was evaluated by immunofluorescence against α-SMA and vWF. The results showed an increasing trend in the number of vessels in the ablation site up to a maximum vascularization level at 150 days; while at the end of the process, with the removal and analysis of the scar at 6 months after ablation (180 days), the data of the healthy and damaged muscle result as very similar, indicating a good vascularization of the scar tissue.

Finally, further immunofluorescence analyses were performed using muscle markers such as myosin heavy chain (MyHC), and matrix marker such as type I collagen (ColI). Comparing the signals obtained from the analyses on the healthy control muscle and the damaged one, a substantial structural and cellular difference was highlighted: the repair tissue was not a muscle tissue because negative for MyHC, while showing positive areas for ColI.

In conclusion we can say that this work confirms the poor muscle tissue regeneration capacity of *Sus scrofa domesticus* upon VML damage and the formation of a vascularized, fibrotic, granulation tissue like in human.

## Conclusions

Muscle ablation at the *Peroneus tertius* in order to generate a porcine model of VML, revealed upon ultrasound, histological and immunofluorescence analysis the formation of fibrous, scar tissue at the damage site. The results showed at later time-points a decrease in inflammation, shown by a reduction of macrophage infiltrate together with an increase in the number of vessels, demonstrating a great scar vascularization.

In conclusion, our work provides a pathophysiological characterization of a long-term repairing process after VML injury into swine, providing a good model to study and better understand the fibrosis during this process. Furthermore, our study confirmed the porcine model effectiveness for the study of future VML injury treatment in humans.

## Materials and methods

### Location and housing condition of pigs

The animals were housed at the Experimental Research Center of Cattolica University of Rome (UCSC), an animal facility authorized by the Ministry of Health, in special newly designed cages to avoid post-surgical problems and for maximum animal comfort, further equipped with environmental enrichment and in accordance with European guidelines 526/2007 and Legislative Decree 26/2014. A veterinary surgeon regularly checked the state of health of the animals and health checks were carried out according to the FELASA guidelines. The housing conditions was suitable for the ethological-behavioral needs of the species in question and meet the requirements of Legislative Decree 26/2014.

### Surgical ablation

Five female specimens two years of *Sus scrofa domesticus*, identified as M6, M7, M8, M9 and M10, were employed in the showed experiments. At the moment of the ablation procedure, the animal weights were respectively: 30,5Kg (M6), 37,5Kg (M7), 36,5Kg (M8), 47Kg (M9) and 32Kg (M10). The pigs were anesthetized with an intramuscular injection of physiological solution (10ml / kg) containing ketamine (5 mg / ml) and xylazine (1 mg / ml). The skin was incised and an opening on the epimysium and the fascia, was created in order to remove a massive portion (about 3 × 2 × 1 cm) of the *peroneus teritus*. After suturing, ultrasound analysis was performed to measure the extension of the damage, calculated as the percentage of the ratio between the damage and the total muscle areas.

We calculated that we took away about 9gr of tissue according to Corona et al. 2018 [45] which measured the correspondence of 1 Volume (cm^3^) of* peroneus tertius* in grams, reporting of the following equation: 1cm^3^ = 1.5gr. The skin was then sutured. After surgery, the animals were subjected to analgesic treatment (Rimadyl, Pfizer, USA) to relieve pain. M6 did not underwent surgical ablation, since it was established as the control in subsequent experiments. The operation was carried out by Dr. Jacopo Baldi, orthopedic surgeon of IFO Regina Elena National Cancer Institute, in a surgical room under the supervision of veterinarian anesthetists and under constant instrumental monitoring of the animal. All experiments with animals were conducted in compliance with current regulations (I.A.C.U.C. n ° 432, 12 March 2006).

### Ultrasound analyses and bioptic sampling

Ultrasound analyses were conducted in collaboration with a team of ecographysts of the University of L’Aquila. In particular, ultrasound checks were carried out at 30, 60, 90, 150 and 180 days after surgical ablation. In order to monitor tissue repair over time, bioptic samples were obtained at the same experimental times as the ultrasound analyses. Sampling was conducted through the ultrasound-guided needle biopsy technique. Briefly, at established time points, a Histocore Kaster biopsy needle was inserted and directed into the tissue of interest thanks to the support of the ultrasound probe in order to obtain “carrots” of tissue with dimensions of 1.8 cm X 0.3 cm. The needle biopsy was performed under local anesthesia in order to avoid pain and suffering to the animals.

### Histological and immunofluorescence analyses

Tissues obtained from the bioptic sampling were accordingly processed for histology and immunofluorescence analyses. Briefly, biopsies were fixed in 4% PFA in PBS for 5 h at 4 °C, rinsed in PBS and left in a solution of 30% Sucrose in ddH_2_O over night at 4 °C. Subsequently, tissues were included in OCT resin (BIO-OPTIC) and quickly frozen in liquid nitrogen to obtain 8 μm-thick sections using a Leica cryostat. Samples were stained with hematoxylin and eosin and Masson’s trichrome (Diapath) staining to have a quick diagnosis of the tissue. Alternatively, samples were analyzed through immunofluorescence as previously described [46]. Briefly, tissues were permeabilized with Triton X-100 0.3% in PBS for 1 h at RT and blocked with a blocking solution consisting of 10% goat serum, 1% glycine, 0.1% Triton X-100 in PBS for 1 h at RT. Subsequently, tissues were incubated with primary antibodies in blocking solution for 1 h at RT and then rinsed with a washing solution consisting of 1% BSA and 0.2% Triton X-100 in PBS. Primary antibodies were diluted as follows: rabbit polyclonal anti-Mannose Receptor (Abcam) 1:100, mouse monoclonal anti-MyHC (MF20, DHSB) 1:50, rabbit polyclonal anti-Collagen I (Abcam) 1:100, mouse monoclonal anti-α-Smooth Muscle Actin (Sigma) 1:100, rabbit polyclonal anti-von Willebrand Factor (Dako) 1:100. After washing, tissues were incubated with Alexa Fluor 555-conjugated goat anti-mouse IgG (H + L; Thermo Fisher Scientific, diluted 1:400) and 488-conjugated goat anti-rabbit IgG (H + L; Thermo Fisher Scientific, diluted 1:400) for 1 h. Finally, nuclei were stained with 300 × 10 − 9 M DAPI (Thermo Fisher Scientific) in PBS for 30 min. Specimens were visualized using a Nikon TE 2000 epifluorescence microscope equipped with a Photometrics Cool SNAP MYO CCD camera and images were acquired through MetaMorph software.

### Digital images analyses

In order to describe the formation of a repair tissue over time, ultrasound images acquired at 30, 60, 90, 150 and 180 days after surgical ablation were analyzed through the ImageJ program. The areas corresponding to either control healthy muscle or to the ablated site were traced through the polygon selections, and the Mean grey value was calculated after selecting the measure of interest in the Set Measurements function (Analyze > Set Measurements > Mean grey value).

### Statistical analyses

All experiments were performed in biological and technical triplicate. Data were analyzed using GraphPad Prism 5, and values were expressed as means ± standard error (SEM). Statistical significance was tested using either ONE WAY ANOVA and Tukey’s post hoc test. A probability of less than 5% (p < 0.05) was considered to be statistically significant.

## Electronic supplementary material

Below is the link to the electronic supplementary material.

Supplementary Materials: Supp. Figure 1. Evaluation of macrophage infiltrate using ImageJ software. Supp. Figure 2. Calculation of the Mean Gray Value starting from ultrasound scans using ImageJ software.
