# Supplementary material for: Long-term longitudinal study on swine VML model
Source: Biol Direct. 2023 Jul 31;18:42. doi: 10.1186/s13062-023-00399-1 (PMC10388508; doi:10.1186/s13062-023-00399-1)
Supplement: Supplementary file 1 — Supplementary Materials: Supp. Figure 1. Evaluation of macrophage infiltrate using ImageJ software. Supp. Figure 2. Calculation of the Mean Gray Value starting from ultrasound scans using ImageJ software. [file 13062_2023_399_MOESM1_ESM.docx]

**Supplementary Materials**

**
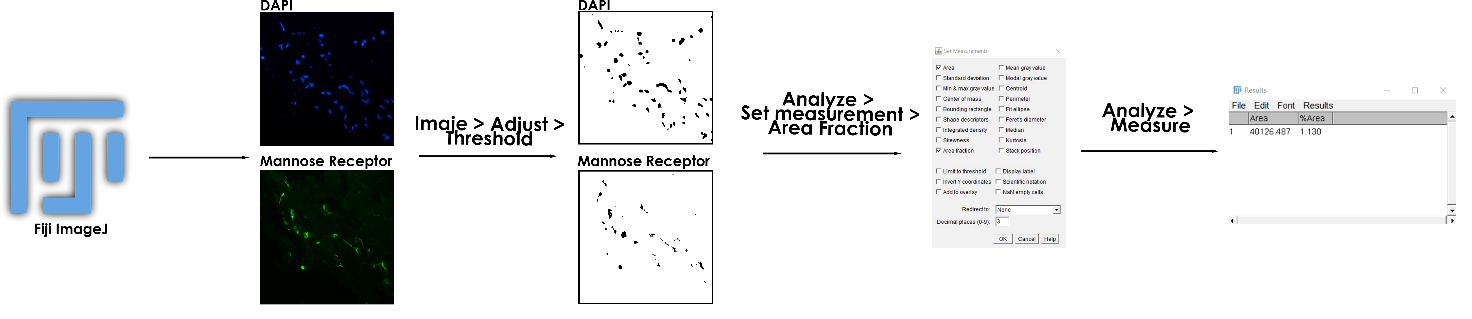
**

**Supplementary Figure 1**. Evaluation of macrophage infiltrate using ImageJ software. Threshold both the nuclei and macrophage images (Image > Adjust > Threshold). Specify in the "Set measurement" section what we want to measure, in this case check "Area fraction" and then click on “Measure” to calculate the positive area. The data must be normalized with the area of the nuclei by making the ratio between the area positive for MR and the area positive for DAPI.

***
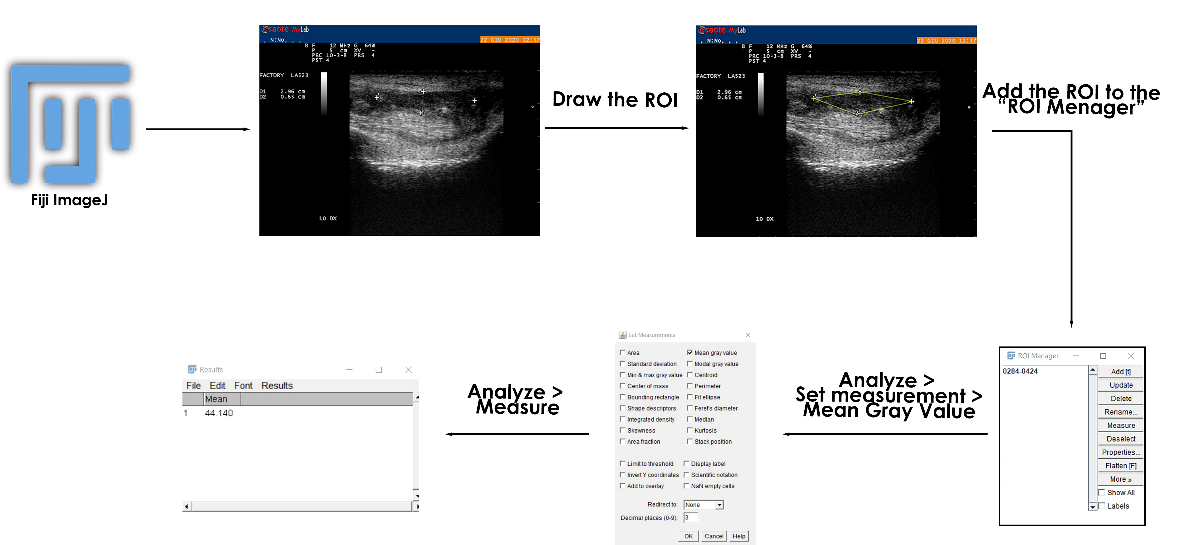
***

**Supplementary Figure 2**. Calculation of the Mean Gray Value starting from ultra-sound scans using ImageJ software. First draw the area of interest on the ultrasound image and add to the "ROI manager". Specify in the "Set measurement" section what we want to measure, in this case check "Mean Gray Value" and then click on “Measure”.
